# Geographical information system and predictive risk maps of urinary schistosomiasis in Ogun State, Nigeria

**DOI:** 10.1186/1471-2334-8-74

**Published:** 2008-05-31

**Authors:** Uwem F Ekpo, Chiedu F Mafiana, Clement O Adeofun, Adewale RT Solarin, Adewumi B Idowu

**Affiliations:** 1 Department of Biological Sciences, University of Agriculture, 110001, Abeokuta, Nigeria; 2 Department of Environmental Management and Toxicology, University of Agriculture, 110001, Abeokuta, Nigeria; 3 Department of Mathematics, University of Agriculture, 110001, Abeokuta, Nigeria

## Abstract

**Background:**

The control of urinary schistosomiasis in Ogun State, Nigeria remains inert due to lack of reliable data on the geographical distribution of the disease and the population at risk. To help in developing a control programme, delineating areas of risk, geographical information system and remotely sensed environmental images were used to developed predictive risk maps of the probability of occurrence of the disease and quantify the risk for infection in Ogun State, Nigeria.

**Methods:**

Infection data used were derived from carefully validated morbidity questionnaires among primary school children in 2001–2002, in which school children were asked among other questions if they have experienced "blood in urine" or urinary schistosomiasis. The infection data from 1,092 schools together with remotely sensed environmental data such as rainfall, vegetation, temperature, soil-types, altitude and land cover were analysis using binary logistic regression models to identify environmental features that influence the spatial distribution of the disease. The final regression equations were then used in Arc View 3.2a GIS software to generate predictive risk maps of the distribution of the disease and population at risk in the state.

**Results:**

Logistic regression analysis shows that the only significant environmental variable in predicting the presence and absence of urinary schistosomiasis in any area of the State was Land Surface Temperature (LST) (B = 0.308, p = 0.013). While LST (B = -0.478, p = 0.035), rainfall (B = -0.006, p = 0.0005), ferric luvisols (B = 0.539, p = 0.274), dystric nitosols (B = 0.133, p = 0.769) and pellic vertisols (B = 1.386, p = 0.008) soils types were the final variables in the model for predicting the probability of an area having an infection prevalence equivalent to or more than 50%. The two predictive risk maps suggest that urinary schistosomiasis is widely distributed and occurring in all the Local Government Areas (LGAs) in State. The high-risk areas (≥ 50% prevalence) however, are confined to scatter foci in the north western part of the State. The model also estimated that 98.99% of schools aged children (5–14 years) are living in areas suitable for urinary schistosomiasis transmission and are at risk of infection.

**Conclusion:**

The risk maps developed will hopefully be useful to the state health officials, by providing them with detailed distribution of urinary schistosomiasis, help to delineate areas for intervention, assesses population at risk thereby helping in optimizing scarce resources.

## Background

Schistosomiasis is a water-borne parasitic disease that affects 200 million people and poses a threat to 600 million in more than 76 countries [1]. It is caused by infection with parasitic worms of the genus *Schistosoma*. These worms are transmitted via contact with contaminated water containing cercaria the infective stage of the parasite [1]. The life cycle of the *Schistosoma haematobium *(the causative agent of urinary schistosomiasis) begins with the excretion of eggs in urine. The eggs hatch in the water and release a free-swimming miracidia whose objective in life is to find and penetrate an appropriate snail (*Bulinus sp*) in which to develop. After a period of asexual reproduction, tailed, free-swimming larvae called cercariae leave the snail and are transported in water where they actively seek and penetrate the skin of humans, thus infecting them [1].

Schistosomiasis is a disease whose distribution is particularly sensitive to environmental changes, including changes of human origin [2]. Transmission of the parasite is focal; whose heterogeneity reflects numerous human and ecological factors [3]. Identifying the broad scale patterns of schistosomiasis is crucial because schistosomiasis control is often diluted at a national level but remains a public health problem in geographically restricted areas [4] and as such, there is a need to identify these areas. Moreover, changing ecology, global warming and migration, may have led to changes in the prevalence and distribution of disease in different parts of the state.

Geographical information system (GIS) and remote sensing (RS) have been used to define the epidemiology of schistosomiasis in many parts of the world [5-10]. Many organizations such as Food and Agriculture Organization (FAO) and United Nations are making RS datasets from earth-observing satellites freely available for researchers through the Internet following increasing recognition that these datasets facilitate the development of powerful tools for disease control [11-13].

Past epidemiological surveys indicate the prevalence of urinary schistosomiasis to be over 80% in some areas in Ogun State, Nigeria [14-17]. However, there exist many areas of the state whose urinary schistosomiasis status remains undefined. Reliable infection prevalence maps that delineate areas of high-risk and quantify population at risk are urgently needed to assist in developing control programme and managing scarce resources. The knowledge of the geographical distribution of the disease through these maps will help guide control efforts. This study therefore provides the first attempts to develop such maps for Ogun State, Nigeria.

## Methods

### Schools survey and infection data

**Figure 1:**
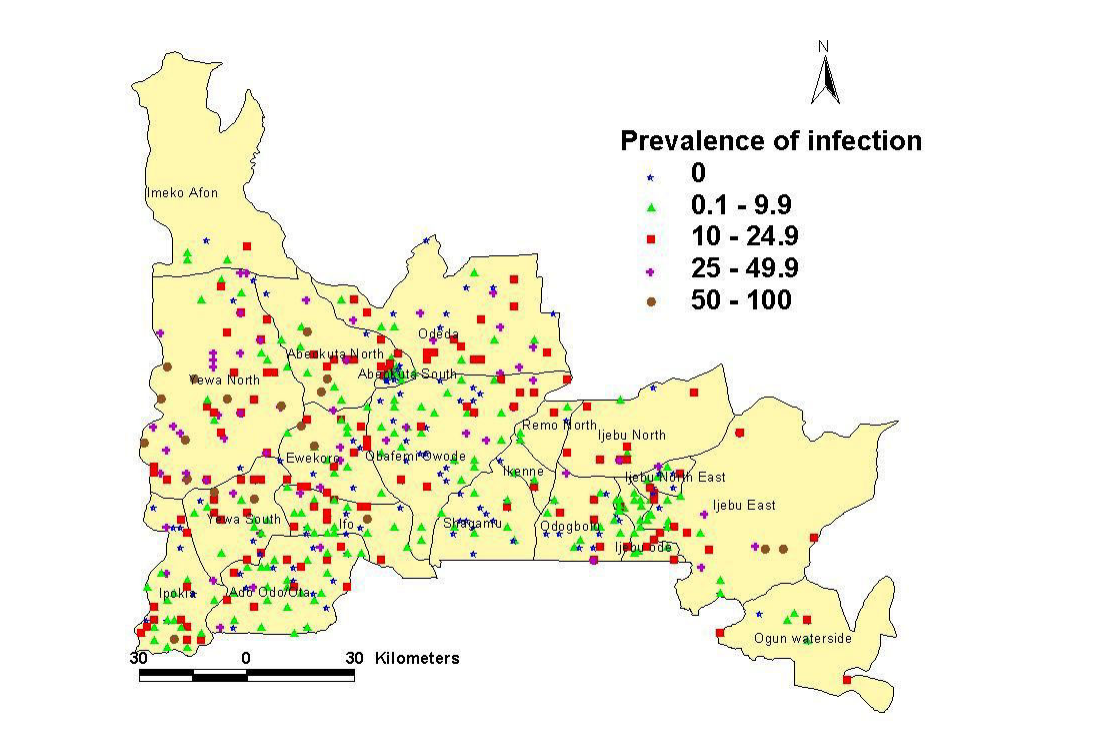
Map of infection prevalence of reported urinary schistosomiasis in Ogun State for 1092 surveyed schools.

Infection data were collected in 2001–2002 using carefully validated school morbidity questionnaires in which school children were asked among other questions, if they had passed blood in urine in the last 3 weeks (*eje ninu ito*) or had urinary schistosomiasis (*atosi aja*). The survey used a stratified random-cluster sampling procedure with the primary school as the basic sampling unit. This was because schoolchildren are the primary targets for treatment and the educational infrastructure can be use to deliver treatment. A total of 101,682 school children from 1,092 schools out of 1,310 schools participated in the survey and formed the basis of our infection data. The questionnaire method was validated in 50 randomly selected schools across the state. In each school, (25 pupils with positive and 25 pupils with negative responses respectively from the question – 'have you passed blood in urine in the last 3 weeks') submitted 2 urine samples each for urine filtration test for *S. heamtobium*. The validation test gave the questionnaire method a sensitivity of 0.88, specificity of 0.71, Negative Predictive Value (NPV) of 0.50 and Positive Predictive Value of 0.95. Spearman rank correlation also showed a highly significant association between reported urinary schistosomiasis and urine filtration test (r = 0.704; p < 0.01). This to our knowledge represents the first ever large scale survey for schistosomiasis in the state. No control programme or mass treatment had been undertaken in any of the schools. The results of survey are shown in Table 1 and the locations of surveyed schools and their point prevalence are shown in Figure 1. The details of the questionnaire, data collection, validation and ethical approval have been reported in a previous publication [18].

**Table 1:** Prevalence of reported urinary schistosomiasis by LGA in Ogun State, Nigeria

LGA	No. of School Surveyed	No. of pupil interviewed	No. of pupil with reported urinary schistosomiasis	Mean Prevalence (95% CI)
Abeokuta North	69	8733	1074	15.40(10.68–20.13)
Abeokuta South	45	6243	118	1.61 (0.83–2.39)
Ado-Odo/Ota	99	10973	581	5.53 (4.29–6.77)
Ewekoro	51	2959	391	13.53 (8.70–18.36)
Ifo	54	6602	361	5.46 (3.46–7.47)
Ijebu East	45	4481	516	10.24 (5.96–14.53)
Ijebu North	38	3124	227	7.54 (4.91–10.18)
Ijebu Ode	30	7706	306	4.73 (2.91–6.55)
Ijebu-North East	39	2032	112	5.54 (2.79–8.29)
Ikenne	20	1743	24	1.46 (0.29–2.63)
Imeko/Afon	37	2563	327	15.47 (10.15–20.80)
Ipokia	57	5035	532	10.88 (7.33–14.44)
Obafemi/Owode	133	6278	535	8.78 (6.52–11.04)
Odeda	67	3739	466	11.38 (7.75–15.01)
Odogbolu	44	3601	178	5.21 (3.20–7.22)
Ogun waterside	49	5234	518	10.49 (6.74–14.24)
Remo North	17	1309	33	2.61 (0.96–4.26)
Sagamu	41	4289	214	3.87 (2.21–5.52)
Yewa North	91	8402	2204	25.07 (21.41–28.73)
Yewa South	66	6636	593	11.76 (8.31–15.21)
Grand Total	1092	101682	9310	10.00 (9.18–10.82)

Reference and sources of data from survey by Ekpo and Mafiana, 2004 [18]

### Remote-sense image and digital environmental data

Land surface temperature (LST) and the normalized difference vegetation index (NDVI) information were derived from the Advanced Very High Resolution Radiometer (AVHRR) on board the National Oceanic and Atmospheric Administration's (NOAA) polar-orbiting meteorological satellites [19] using standard procedures [20]. Image data for 2001–2002 were processed by means of unsupervised classification procedures of Earth Resources Data Analysis System (ERDAS) Imagine 8.4™ (Leica Geosystems, ERDAS Inc. Atlanta, GA, USA) software. Minimum, mean, and maximum values of these data were extracted for each pixel that corresponded to the location of the schools [21]. Soil-types, land cover and interpolated rainfall and altitude maps of study area were obtained courtesy of GIS unit, International Institute for Tropical Agriculture (IITA), Ibadan. The data came already geo-referenced in geographic (plane) projection. Using cross-tabulation module of ArcView GIS spatial analyst, altitude, rainfall, soil type and land cover values corresponding to location of study schools were derived.

### Population data

The population of school age children (5–14 years) in each Local Government Area was obtained from the 1991 National Population Census and projected to 2002 using Ogun State Government annual specific growth rate of 2.83% from National Population Commission [22] with additional demography information from [23]. This age-group is most likely to have schistosome infection and is consequently largely responsible for maintaining transmission in endemic areas.

### Location of schools

Coordinates (latitude/longitude) of schools were collected in the field using a Garmin 12XL (Garmin Corporation, USA) Global Positioning System (GPS) during the survey [18].

### Data analysis

Analysis of relationships between school infection prevalence and environmental data involved logistic regression. An initial model was established, defining *S. haematobium *infected schools as cases, and incorporating NDVI, LST, rainfall, soil-type, land cover and altitude variables as covariates. The non-significant associations were removed with a forward step-wise elimination technique [21]. The same procedure was also used in a second model where school prevalence of *S. haematobium *was dichotomised at ≥ 50% level as high-risk. For those associations that remained significant, their odds ratio (including 95% confidence intervals), the likelihood ratio, and *P *values were calculated.

### Regression calculations

The relationship may be model as follows:

(a) Probability of presence of urinary schistosomiasis in a school is given by the following logistic regression equation:

Probability (P) = 1/(1 + e ^-z^),

Arising from the logistic regression analysis shown in Table 2, 3, and 4, z is calculated as follows:

**Table 2:** Coefficients and goodness of fit of a logistic binary model predicting presence and absence of urinary schistosomiasis in different schools of Ogun State, on the basis of observed data of disease and environmental variables: Percentages of correct predictions.

Observed Urinary Schistosomiasis	Predicted Urinary Schistosomiasis		Correct prediction (%)
		
	Absent	Present	
Infection Absent	0	136	0.0
Infection Present	0	553	100.0
Overall			79.7

**Table 3:** Coefficients and goodness of fit of a logistic binary model predicting presence and absence of urinary schistosomiasis in different schools of Ogun State, on the basis of observed data of disease and environmental variables: Variables remaining in the equation

Variable	B	SE	Wald	df	Sig.	Exp (B)	95% C.I. for Exp (B)	
							
Model 1							Lower	Upper
LST	0.308	0.124	6.209	1	0.013	1.361	1.068	1.734
Constant	-7.766	3.659	4.505	1	0.034	-	-	-

*df = degree of freedom; SE = standard error; Sig = significance; B = regression coefficient; Exp(B) = odd ratio. Note: Table shows final Model after forward stepwise elimination of non-significant variables.

**Table 4:** Coefficients and goodness of fit of a logistic binary model predicting presence and absence of schistosomiasis in different schools of Ogun State, on the basis of observed data of disease and environmental variables: Model with terms removed

Model step	Term removed	Model Log Likelihood	Change in -2log Likelihood	df	Significance of change
Final	LST	-337.797	6.376	1	0.012

Z = -7.766 + (0.308 × LST) where

-7.766 is the regression coefficient constant, and 0.0308 is the regression coefficient of LST.

(b) Probability of an area having infection prevalence > 50% (high-risk) is given by the following logistic regression equation:

Probability (P) = 1/(1 + e ^-z^),

Arising from the logistic regression analysis shown in Tables 5, 6 and 7: z is calculated as follows:

**Table 5:** Coefficients and goodness of fit of a logistic binary model predicting the probability of high-risk schools for urinary schistosomiasis in Ogun State, on the basis of observed data of disease and environmental variables: Percentages of correct predictions.

Observed Urinary Schistosomiasis	Predicted Urinary Schistosomiasis		Correct prediction (%)
		
	Absent	Present	
Low-risk	456	2	99.6
High-risk	69	6	8.0
Overall			86.7

**Table 6:** Coefficients and goodness of fit of a logistic binary model predicting the probability of high-risk schools for urinary schistosomiasis in Ogun State, on the basis of observed data of disease and environmental variables: Variables in the equation.

Variable	B	SE	Wald	df	Sig.	Exp (B)	95% C.I. for Exp (B)	
							
Model 2							Lower	Upper
LST	-0.478	0.227	4.441	1	0.035	0.620	0.397	0.967
Rainfall	-0.006	0.002	16.318	1	0.0005	0.994	0.991	0.997
Soiltype			11.706	3	0.008			
Soiltype (1)	0.539	0.493	1.196	1	0.274	1.714	0.653	4.503
Soiltype (2)	0.133	0.451	0.086	1	0.769	1.142	0.472	2.765
Soiltype (3)	1.386	0.523	7.015	1	0.008	3.998	1.434	11.151
Constant	20.593	8.254	6.225	1	0.013	8.8 × 10^8^		

*df = degree of freedom; SE = standard error; Sig = significance; B = regression coefficient; Exp(B) = odd ratio.

Note: Table shows final Model after forward stepwise elimination of non-significant variables.

**Table 7:** Coefficients and goodness of fit of a logistic binary model predicting the probability of high-risk schools for urinary schistosomiasis in Ogun State, on the basis of observed data of disease and environmental variables: Model with terms removed.

Term removed	Model Log Likelihood	Change in -2log Likelihood	df	Significance of change
LST	-194.831	4.482	1	0.034
Rainfall	-202.552	19.924	1	0.0005
Soil types	-198.316	11.452	3	0.010

df = degree of freedom

Z = 20.593+ (-0.478 × LST) + (-0.006 × rainfall) + (0.539 × ferric luvisols (soil type 1) + (0.133 × dystric nitosols (soil type 2) + 1.386 × pellic vertisols (soil type 3)

### Predictive GIS risk model maps

Using the image calculator module the spatial analysis extension of ArcView version 3.2a (ESRI, Redland, CA), the logistic regression models obtained from the analysis were applied to the raster data sets of variables in the finals models i.e. LST, soil types and rainfall to generate predictive model maps of urinary schistosomiasis infection in the state.

### Estimation of school-aged population at risk of urinary schistosomiasis

An arbitrary criterion based on whether the logistic regression probability of finding an infection is ≥ 0.75% within any area was used. On this basis, the number of school-aged children at risk of significant schistosomiasis transmission was quantified by overlaying the predictive model map of presence or absent of infection developed on population database for school-aged (5–14 years) children in the State to create a suitability risk map. The total numbers of school-aged children at risk were then extracted for each LGA using cross-tabulation module in ArcView GIS 3.2a [24].

## Results

### Predictive GIS risk model maps

The logistic regression analysis showed that the significant variable in predicting the presence and absence of urinary schistosomiasis in any school in the State was mean minimum LST.

The estimated coefficients (the intercept), the standard errors, and the goodness of fit of the binary models are shown in Table 2, 3, and 4. The model correctly predicted all the 553 observed positive schools as schistosomiasis infected schools. Although none of the 136 negative schools were correctly predicted by the model to be free of infection, there was 79.7% overall accuracy of the model in predicting infected and non-infected schools.

In identifying high and low-risk areas, LST, rainfall, and three soil types: ferric luvisols, dystric nitosols and pellic vertisols soils were the final variables in the model for predicting the probability of an area having infection prevalence (equivalent of > 50% parasitological prevalence). Only the pellic vertisols soil type was significant.

The estimated coefficients, the standard errors, and goodness of fit of the binary model are shown in Table 5, 6, and 7. The model correctly predicted 456 (99.6%) of all observed low-risk schools as low-risk and 6 (8.0%) of high-risk school. There was 86.7% overall accuracy of the model in predicting low and high-risk schools.

**Figure 2:**
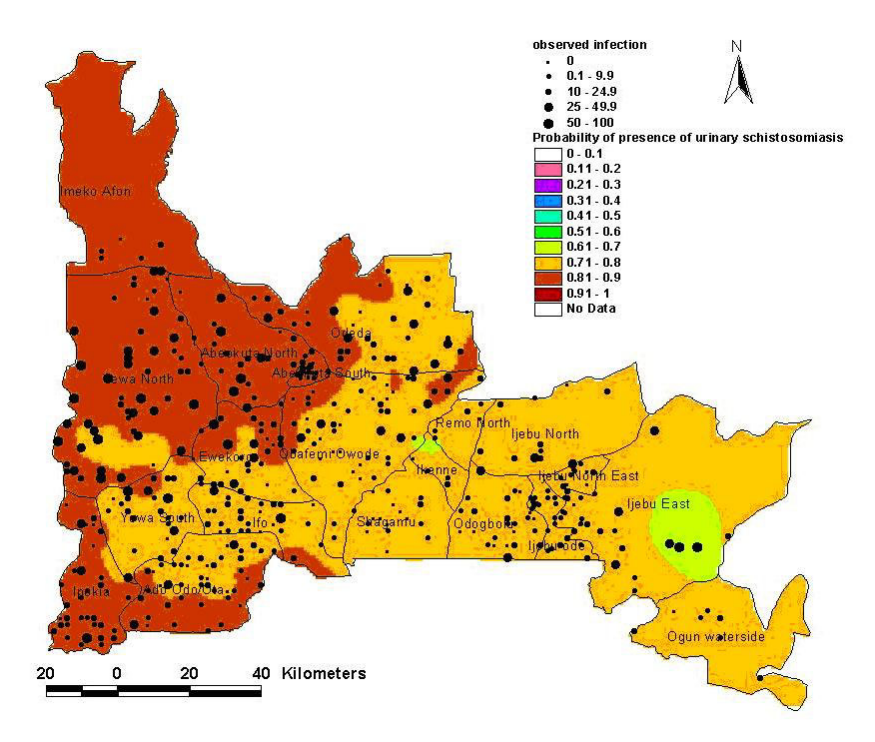
Risk model map of presence of urinary schistosomiasis in Ogun State as observed and predicted through logistic regression.

**Figure 3:**
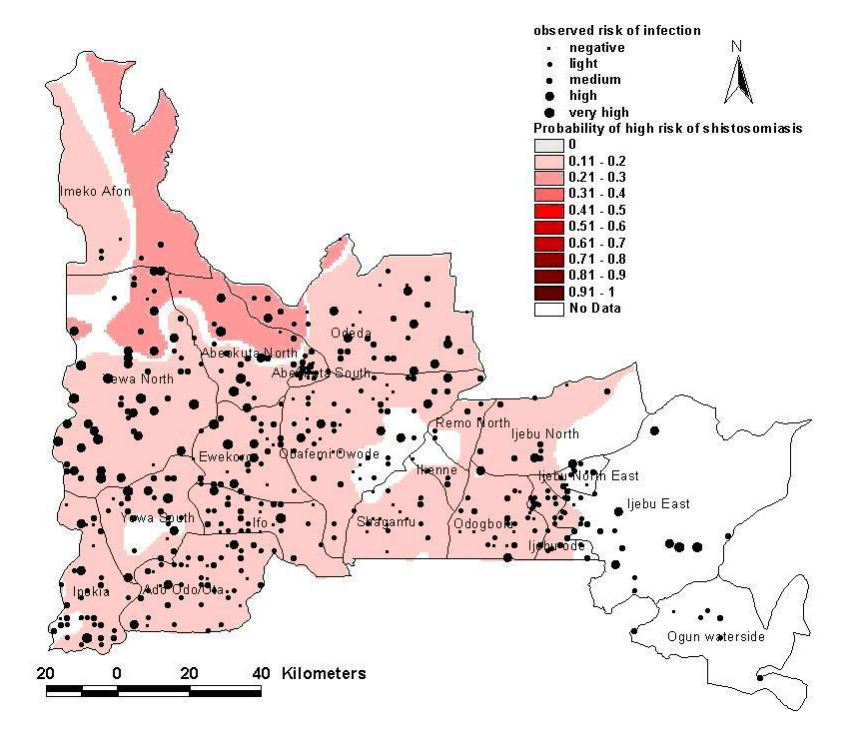
Risk model map of presence of high-risk schools for urinary schistosomiasis in Ogun State as observed and predicted through logistic regression.

The resulting risk model maps are shown in Figures 2 and 3. The first model showed that risk for infection of urinary schistosomiasis increases in the south-westerly direction, with places of maximum risk (0.8–1.0) of finding infected schools in the following LGAs: Imeko/Afon, Yewa North, Abeokuta North, Abeokuta South, parts of Odeda, Obafemi/Owode, Ewekoro, Ipokia, Ifo and Ado-Odo/Ota. Medium risk areas include most of Odeda, Obafemi/Owode, Yewa South; parts of Ado-Odo/Ota, Ifo, Sagamu, Ijebu axis, Areas with less risk of finding infected schools are confined to foci in Ikenne and Ijebu East. The second model shows that high-risk schools with urinary schistosomiasis are confined to the northwest axis of the state, comprising parts of the following LGAs: Imeko/Afon, Abeokuta North, Odeda and Yewa North. The predicted probability of finding high-risk schools was less than 0.4.

### School-age population at risk of infection

**Figure 4:**
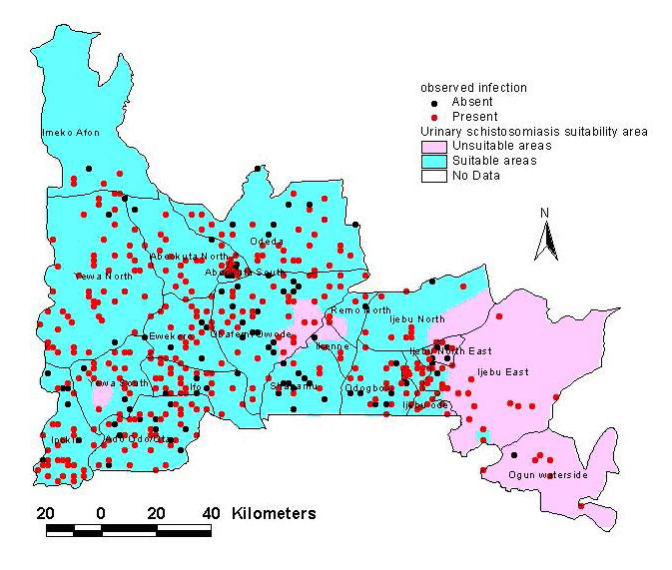
Risk map of suitable areas for urinary schistosomiasis transmission in Ogun State based on predicted probability of 0.75.

Superimposing the population density map for school age children living in Ogun State with grid image of probability of infection derived from model 1, an image of suitability risk area was created (Figure 4). The estimated totals extracted from the map indicated that out of 809,222 school aged children living in Ogun State, 801,075 (98.99%) are living in areas that can harbour the infection.

## Discussion

Like many other diseases, urinary schistosomiasis has a "natural habitat" [25], hence its distribution and prevalence are greatly influenced by environmental factors affecting the population of snail intermediate hosts, and human hosts. Although this notion has long been realized, and despite the expansion of information on effects of environmental factors on snail intermediate host of urinary schistosomiasis [26], little attempt has been made to map the distribution of urinary schistosomiasis in relation to specific environmental factors in Nigeria. Such maps would allow control programme managers to define the extent of the problem and use intervention rationally, where it is most likely to succeed. Schistosomiasis is known to occur in many areas in the country [1]; however, there has not been any sustained effort to control the disease, apart from the establishment of the National Schistosomiasis Control Programme in 1988. It has been difficult for the programme to make measurable progress due to the lack of baseline data and detailed information of the geographic distribution of schistosomiasis. This situation has not only hampered drug procurement, drug distribution and programme evaluation but also led to difficulties in obtaining funding from the government and donor agencies for controlling the infection.

Presented in this study are the first risk-maps of the predicted distribution of urinary schistosomiasis in Ogun State. Although, attempt to validate the models has not been done due to paucity of funds. None the less, this study has provided baseline data that can be used in the context of controlling the disease. The present analysis depends on self-reported urinary schistosomiasis from school children which has been shown to offer cheap, rapid and reliable way of urinary schistosomiasis risk estimate [27-30]. In all the logistic regression analyses carried out in this study, LST appeared to be the most important predictive variable affecting both the probability of presence and the actual prevalence of the disease. The significance of temperature in the distribution of schistosomiasis has also been reported in past studies using GIS in East and South Africa [21,24]. Altitude and NDVI did not correlate with presence or prevalence of urinary schistosomiasis in Ogun State. These results are contrary to the observations using these environmental variables in Tanzania and Egypt [21,31], where altitude and NDVI were shown to be important environmental variables in predicting the distribution of schistosomiasis. The reason for this may be that there are little or no variations in altitude and NDVI values in Ogun State to significantly affect the distribution of urinary schistosomiasis. Altitude affects both temperature and rainfall, and serves to restrict the distribution of snail species by providing an upper and lower limit for population and transmission dynamics [32]. Altitude and NDVI however, has been shown to be an important environmental variable in predicting the distribution of schistosomiasis in Tanzania, Egypt, and Ethiopia where there is great variation of these variables due to the climate and land mass [32]. NDVI was also used in mapping the distribution of snail intermediate host in China [33]. Another explanation may be the small spatial scale of the study area, which is Ogun State as its extent is small when compared to the whole of Tanzania, Egypt and China. Perhaps the application of these variables on a broader scale such as the whole of Nigeria may provide results different from this study.

Current estimates of the number of school aged children at risk of urinary schistosomiasis in Ogun State are lacking as this is the highest risk group. This study has estimated population of school aged children (5–14 years) at risk from urinary schistosomiasis in Ogun State to be 801075 (0.80 million) as at year 2002. However, it should be appreciated that this estimates represents school-aged populations living in areas where the environmental factors when combine with host/parasite are suitable for infection transmission. It does not necessarily imply the number of school-aged children infected. The development of infection suitability maps in this study suggests that 98.99% of school children in the State are living in schistosomiasis suitability area and therefore at risk of urinary schistosomiasis. The model predicts that except for few foci, all the school children in the state are living in urinary schistosomiasis areas where transmission is suitable or ongoing. This is first of such model for Ogun State. This observation is important and has implication for water resources development such as dam construction and irrigation where recent estimation put the population of the country at risk to be 101.28 million [34]. There will be a need for Ogun State Government to establish a schistosomiasis risk management agency to monitor any water development project such as irrigation that may increase the risk of schistosomiasis transmission. With 50% of the LGAs having 100% risk of infection, the agency could also develop intervention for at risk area. A classical example of lack of intervention measures was the outbreak of urinary schistosomiasis in communities near Oyan dam following its construction in 1984 in Abeokuta North LGA, Ogun State [35], whose transmission remain unabated till date [36].

Thus these maps will provide the state with information on which areas to include in treatment programme. The maps developed in the study can also be used to plan risk assessment and monitoring of urinary schistosomiasis control. This method offers a rapid approach for mapping the distribution of diseases in others States in the country and for estimating the population at risk as a first step in reactivating the control of this neglected tropical disease. It will be useful to improve on the model and maps by including *Bulinus *snail intermediate host distribution data as well as analysing for, if any, spatial correlation in the questionnaire data used, given the focal nature of schistosomiasis. With funding, it would be possible to expand this kind of studies to include the whole country of Nigeria and thus produce a reliable base for developing a well-adapted national schistosomiasis control programme.

## Conclusion

The predictive risk model maps developed in this study has provided detailed mapping of the geographical distribution of urinary schistosomiasis in Ogun State. The fact that the models were derived from environmental variables plus prevalence survey data enabled us to map the disease and predict its burden across the State. These maps would be of great value for planning treatment and intervention programme in the State.

## Competing interests

The authors declare that they have no competing interests.

## Authors' contributions

UFE conceived the study, analysis and developed the GIS analysis protocols. CFM provided logistic advice and supervised the analysis. COA helped in interpretation of remotely-sensed image. ABI and ARTS undertook the development of the statistical models to be used in GIS. All contributed to the final report.

## Pre-publication history

The pre-publication history for this paper can be accessed here:


